# Familial Risk Factors and Emotional Problems in Early Childhood: The Promotive and Protective Role of Children’s Self-Efficacy and Self-Concept

**DOI:** 10.3389/fpsyg.2020.547368

**Published:** 2020-11-16

**Authors:** Fabio Sticca, Corina Wustmann Seiler, Olivia Gasser-Haas

**Affiliations:** ^1^Marie Meierhofer Children’s Institute, Associated Institute of the University of Zurich, Zurich, Switzerland; ^2^Department of Pre-Primary and Lower Primary Level, Zurich University of Teacher Education, Zurich, Switzerland

**Keywords:** familial risk factors, emotional problems, self-efficacy, self-concept, longitudinal, early childhood, promotive, protective

## Abstract

The present study aimed to examine the promotive and protective role of general self-efficacy and positive self-concept in the context of the effects of early familial risk factors on children’s development of emotional problems from early to middle childhood. A total of 293 (T1; M_age_ = 2.81), 239 (T2; M_age_ = 3.76), and 189 (T3; M_age_ = 9.69) children from 25 childcare centers took part in the present study. Fourteen familial risk factors were assessed at T1 using an interview and a questionnaire that were administered to children’s primary caregivers. These 14 familial risk factors were used to compute a familial risk factors score. Primary caregivers also reported on their children’s emotional problems at T2 and T3 and on their children’s general self-efficacy at T2. Children reported on their positive self-concept at T2. Results showed that early familial risk factors were positively associated with emotional problems in the short and long term, although the long-term effect was small and non-significant. Further, the pattern of effect sizes of both promotive and protective effects of general self-efficacy as well as positive self-concept was found to be consistent in the short term. However, in the long term, no consistent support for either the promotive or the protective role of general self-efficacy or positive self-concept was found. These results suggest that general self-efficacy and positive self-concept might contribute to promote mental health and to protect from undesired effects of familial risk factors in the short term. Possible reasons for a lack of long-term effects are discussed along with practical implications.

## Introduction

Exposure to adversity during the first years of life can have a detrimental impact on children’s development (Shonkoff and Garner, 2012; Slavich, 2016; Bright and Thompson, 2018). A rich body of literature has identified an extensive array of potential early threats for healthy child development as well as an equally broad spectrum of undesirable outcomes for children (Wright et al., 2013). At the same time, numerous factors have been identified that promote healthy development and/or protect from undesirable outcomes (Rutter, 1987; Wright et al., 2013; Masten, 2015). One aspect of this complex interplay that has yet to be examined is the short- and long-term promotive and protective role of children’s self-referential mental representations. Given the prominent role of the familial context in early childhood (Masten and Barnes, 2018), the aim of the present study was to address this research gap with a focus on the longitudinal association between familial risk factors and children’s emotional problems from early to middle childhood.

Familial risk factors are characteristics of family members as well as attributes of the family as a whole. Examples of familial risk factors are (a) socio-economic aspects such as familial poverty, low parental education, or single parent family (e.g., Jasiulione and Jusiene, 2019), (b) interpersonal aspects such as familial conflict, maltreatment, or abuse (e.g., Keil et al., 2018), (c) critical life events such as death or illness of significant others, frequent move, or migration (e.g., Høeg et al., 2018), and (d) other risks including parental drug abuse or parental mental illness (e.g., Ahun and Côté, 2019).

Early familial risks can have detrimental effects on a wide range of developmental outcomes. Potential outcomes encompass brain development, cognitive development, academic development, language development, and emotional development (e.g., Gerhardt, 2006; Sylvestre and Mérette, 2010; Fay-Stammbach et al., 2014; Appleton et al., 2017; Mall et al., 2018; Sattler and Gershoff, 2019). A number of common findings regarding the way that familial risk factors affect child development have been identified (Masten and Barnes, 2018). First, the cumulative amount of exposure to risk is positively associated with the probability of undesirable outcomes (Garmezy, 1993). Second, the timing of exposure to the risk and its coincidence with the timing of developmental processes and critical life events (e.g., transitions) is crucial (Cavanagh and Huston, 2008). Third, there are constellations in which exposure to adversity has a strengthening effect on the individual experiencing the adversity (i.e., “steeling effects”; Rutter, 2012). Fourth, undesirable effects of risks can be found even after prolonged time periods, but tend to diminish with time (Korol et al., 2002). In contrast, risk factors and their interaction might create a chain reaction that leads to undesirable long-term consequences (Moffitt, 1993). Last but not least, the same level of exposure to adversity can have very different consequences on different individuals (Dutra-Thomé et al., 2018), for instance as a function of age and/or gender (Donders and Woodward, 2003; Fix et al., 2019), which is known as the phenomenon of differential susceptibility.

One of the most robust findings of research on early exposure to adversity is that children respond differently to comparable amounts of risk (Bonanno and Diminich, 2013). This differential susceptibility is believed to stem from the diversity among individuals both as a complex organism and as a part of an equally complex system (Masten and Barnes, 2018). In accordance with developmental system theory (Ford and Lerner, 1992), Masten (2011) conceptualized resilience as the ability of a system to maintain healthy functioning in the face of adversity. Thus, resilience is understood as a multi-layered process among multiple interacting variables of different levels of the system (Chmitorz et al., 2018). These variables can be ordered into risk, promotive, and protective factors. Risk factors are variables that increase the likelihood of unfavorable outcomes (see above). Promotive factors have favorable effects independently of the amount of risk. Finally, protective factors progressively unfold their protective effects as a function of the amount of risk. While a factor can be promotive and protective at the same time (Masten and Barnes, 2018), it is also possible that it is promotive only (Burke et al., 2017) or protective only (Wustmann Seiler et al., 2017).

The present study focused on examining the promotive and protective role of self-referential mental representations, namely general self-efficacy and positive self-concept, in the link between familial risk factors and emotional problems. Masten and Barnes (2018) summarized and discussed the finding from decades of research about promotive and protective effects in various disciplines and identified a set of common resilience factors for child development. One element of the short-list of these factors was self-efficacy and positive views of the self or identity. Both of these constructs are linked to the way individuals address and process the outcomes of both everyday events as well as challenging and stressful events (Shavelson et al., 1976; Bandura, 1977; Scott et al., 2008). For instance, individuals with high levels of self-efficacy tend to believe more in having control over the outcomes of their actions but they also tend to evaluate these outcomes in a more positive way (Ozer and Bandura, 1990), which has a positive effect on their emotional well-being. The state of research regarding the link between self-efficacy as well as self-concept with emotional problems and related constructs is outlined in the following with a focus on protective and promotive mechanisms.

Bandura (1977, p. 193) defined efficacy expectations (i.e., perceived self-efficacy expectations) as “the conviction that one can successfully execute the behavior required to produce the outcomes.” Further, he defined three dimensions of self-efficacy expectations: Magnitude, strength, and generality. Magnitude refers to the difficulty of tasks, while strength refers to how high the expectation of success is, and generality refers to how broad the effect of mastering a task is on self-efficacy expectations. While mastery experiences might specifically increase the self-efficacy for a given task, other experiences might have spillover effects and increase the general level of self-efficacy. Therefore, it can be assumed that individuals differ in their level of trait-like “general self-efficacy” (Schwarzer and Warner, 2013).

Self-efficacy has been found to be central at various levels of a system such as, for instance, a school, a family, or an individual (e.g., Bandura et al., 1999; You et al., 2016; Höltge et al., 2019). Indeed, it was shown to be positively associated not only with performance related constructs like coping behavior, problem solving, and academic performance (e.g., Caprara et al., 2008; Ebner et al., 2018; Li et al., 2018), but also with indicators of mental health such as lower anxiety and depression, as well as higher life satisfaction (e.g., Bandura et al., 1999; Tahmassian and Moghadam, 2011; Moksnes et al., 2018). Results from large-scale studies on resilience showed that self-efficacy is positively associated with resilience (Werner, 1996; Masten et al., 1999). In one of the few studies with preschool-aged children, Miller-Lewis et al. (2013) reported that a composite score of self-concept consisting of self-efficacy and self-worth had a promotive but no protective effect on parent-reported child mental health. Notably, most of the research in this area focused on the self-efficacy of adolescents and adults, while little research on the role of children’s self-efficacy for resilience processes has been conducted in the context of *early* childhood.

Studies on the role of self-efficacy for the longitudinal development of depression in older children support its link to emotional problems. Bandura et al. (1999) were able to show that academic and social self-efficacy were both directly and indirectly linked to depression as well as changes in depression over time in early adolescence. Similarly, Caprara et al. (2010) were able to identify a complex longitudinal indirect link between affective, filial, and resistive regulatory self-efficacy and depression across adolescence. Also in line with these findings, Steca et al. (2014) found that academic and social self-efficacy were negatively related to depressive symptoms in middle childhood. Further, the authors reported that effects of changes in hassles on changes in depressive symptoms were positive on average but were less pronounced with increasing scores of self-efficacy, which indicated that self-efficacy had a protective effect. While these studies suggest that self-efficacy is linked to emotional problems, the question about its promotive and protective role in early and middle childhood remains unanswered. Understanding the potential contribution of self-efficacy as a promotive and/or protective factor is essential given that, besides being a public health issue on its own, childhood depression can have long-lasting effects on well-being up to adulthood (Petersen et al., 1993).

As with self-efficacy, self-concept is a very well studied construct, particularly in educational psychology (Möller and Marsh, 2013). Shavelson et al. (1976) defined self-concept as “a person’s perception of himself” and described it as organized, multifaceted, hierarchical, stable, developing, evaluative, and differentiable. Besides its prominent role in educational psychology, self-concept also been found to be linked to indicators of mental health, including for instance anxiety, loneliness, self-perceived health, and quality of life (Zissi et al., 1998; Park, 2003; Sahranavard, 2014; Xu and Chen, 2018).

Studies on the role of children’s self-concept for resilience processes in early childhood with a focus on emotional problems could not be found. Nonetheless, results from studies with older subjects generated important knowledge that might also apply to younger children. Emerson et al. (2019) found that decreases in depressive symptoms were linked to increases in self-concept in adolescents with a chronic illness that participated in a psychosocial, family based intensive outpatient program. Chavez-Hernandez et al. (2018) reported that self-evaluation and family self-concept were associated with depression in middle to late childhood. Similarly, Kuzucu et al. (2014) found that depression was negatively linked to self-concept development from late childhood to adolescence. In the academic context, Wu and Kuo (2015) found that self-concept acted as a mediator in the link between academic achievement and depression, especially among children in grades 3–4 as compared to grades 5–6. In another study that conceptualized self-concept as a mediator, Spilt et al. (2014) were able to show that self-concept longitudinally mediated the effect of peer rejection on internalizing symptoms. Regarding studies that conceptualized self-concept as a moderator or a protective construct, Jaureguizar et al. (2018) described that children’s self-concept cross-sectionally buffered the positive effect of self-reported stress on teacher-reported depression in late childhood. In this study, self-concept was treated as a moderator together with resilience and social skills, which shows that not only self-efficacy (see above) but also self-concept is conceptually handled as an indicator of resiliency in some frameworks. Nguyen and Scott (2013) found that physical self-concept and English self-concept (but not mathematics self-concept) buffered the negative effect of the death of a family member among 5th-graders. These studies indicate that self-concept is linked to depressive symptomatology. Nevertheless, as with self-efficacy, the question about its promotive and protective role in early and middle childhood has yet to be enlightened.

The aim of the present study was to examine the short- and long-term promotive and protective role of general self-efficacy and positive self-concept in the context of the effects of early familial risks on children’s development of emotional problems. As far as the short-term perspective is concerned, we hypothesized that (H1) familial risks at T1 would be positively associated with emotional problems at T2, that (H2a) self-efficacy as well as (H2b) positive self-concept at T2 would be negatively linked to emotional problems at T2 (i.e., promotive effects), and that the effect of familial risks on emotional problems at T2 would be negatively moderated by self-efficacy (H3a) and positive self-concept (H3b; i.e., protective effects). Given the paucity of longitudinal research in this area, we did not formulate any specific research questions for the long-term effects, although from a theoretical point of view, we expected that the same pattern of results formulated above would apply to the longitudinal perspective as well.

## Materials and Methods

### Sample and Procedure

A sample of 25 childcare centers from the German-speaking part of Switzerland were recruited for study participation. In Switzerland, childcare centers are often composed of multiple small childcare groups. All 63 childcare groups of these childcare centers participated in the study. A total of 293 Children (M_age_ = 2.81; *SD*_age_ = 0.55; 47.9% female) and their primary caregivers participated at T1 in 2009. In 2010, the same children (M_age_ = 3.76; *SD*_age_ = 0.49; 47.3% female) and their primary caregivers were enrolled for participation in T2. The participation rate was 81.5% (i.e., 239 children). Finally, in 2016, 189 children (M_age_ = 9.69; *SD*_age_ = 0.48; 48.6% female) and primary caregivers took part in a long-term follow up study T3 (i.e., 79.0% of those participating at T2). The sample consisted mainly of highly educated primary caregivers: The percentage of primary caregivers who had at least a university degree was 63.1%, 64.8%, and 69.5% at T1, T2, and T3, respectively. Further 89.2% of primary caregivers had a Swiss nationality and 84.2% spoke the local language at home (i.e., Swiss German).

### Ethical Procedure

Primary caregivers were informed about the aims and procedures of the study and gave their written consent for the procedure describe within a written project description that was handed out beforehand. Both primary caregivers and children were also informed about their right to quit their participation at any time without having to indicate any reason and were informed that data would be stored on a secured server in Switzerland in anonymized form and that it would exclusively be used for research purposes at the Marie Meierhofer Children’s Institute. Children were given a small gift after each assessment. All procedures were in line with the Swiss legislation and no ethical approval was needed according to the Swiss legislation, as confirmed by the ethics committee of the canton of Zurich.

### Study Measures

#### Familial Risk Factors

In order to reduce the occurrence of socially desired response patterns, a primary caregiver interview was used to address the less sensitive familial risk factors while a paper and pencil questionnaire was used to assess the most sensitive ones. Both instruments were administered at T1. The interview protocol was adapted from various instruments that were used in research on familial risk factors (e.g., Rutter and Quinton, 1977; Esser et al., 1989) and was administered by trained undergraduate students following a standardized procedure. Herein, our aim was to assess a wide array of familial risk factors in order to derive a cumulative risk score. The choice of this approach was based on theoretical as well as methodological considerations. From a theoretical point of view the cumulative risk hypothesis (for a review, see Evans et al., 2013), postulates that while single risk factors can have an impact on child development, the accumulation of such risk factors is more meaningful as a predictor of child development than single risk factors (e.g., Appleyard et al., 2005). From a methodological point of view, cumulative risk factors often perform better in predicting child development outcomes than single indicators (e.g., Hancock et al., 2018). Further, the cumulative risk factor is better suited to capture the effects of multiple risk factors without overloading statistical models, particularly in studies working with small samples. Nonetheless, it is important to acknowledge, that the use of cumulative risk scores comes with several drawbacks: Evans et al. (2013) highlighted that besides the choice of risk factors being commonly atheoretical, the cumulative approach assumes that risk factors have additive effects only, while their effects are more likely to be multiplicative. While this limitation is important, the authors pointed out that examining the interaction effects of multiple risk factors becomes very cumbersome and susceptible to multicollinearity. In the present study, a total of 14 familial risk factors were included for the purpose of the analyses. Accordingly, our aim was to model a cumulative familial risk score that encompassed both distal and proximal as well as broad and narrow risk factors. The operationalization as well as the conditions that were set to decide about the presence of a risk factor are reported in Table 1 together with the respective frequencies. The mean score of these 14 dichotomous or dichotomized familial risk factors was computed to obtain an overall score of familial risks.

**TABLE 1:** Operationalization of all indicators of familial risk factors.

**Indicator of familial risk**	**%**	**Type**	**Condition for a code of 1 (i.e., child is exposed the respective risk factor)**
Single parent family	10.0	Dich.	M or F reports being single.
Alcohol and/or drug abuse of M and F	5.4	Dich.	M and F report using alcohol and/or other drugs.
Current and/or previous family violence	3.4	Dich.	M and/or F reports that the child witnessed violence between caregivers.
Current and/or previous chronic partnership disharmony	10.7	Dich.	M and/or F reports that the child witnessed long lasting verbal conflicts between caregivers.
Family income below poverty threshold	12.7	Ord.	M and F report that their household income is below the poverty threshold of CHF 5200 per month.
Low maternal education	7.9	Ord.	M reports having a highest academic degree of primary or secondary level.
Immigrant background of the family	16.8	Dich.	M and/or F reports that their family language differs from the local language.
Serious illness or death of a primary caregiver	3.0	Dich.	M and/or F reports that a primary caregiver died or suffered from a serious illness in the last 12 months.
Serious illness or death of another family member	2.7	Dich.	M and/or F reports that a family member other than a primary caregiver died or suffered from a serious illness in the last 12 months.
Serious illness or death of a friend	1.0	Dich.	M and/or F reports that a child’s close friend died or suffered from a serious illness in the last 12 months.
Serious illness of a sibling	4.1	Dich.	M and/or F reports that a sibling suffers from a serious illness.
Self-reported mental health issues of M and/or F	4.9	Dich.	M and/or F reports that she/he is not doing well in terms of subjective mental health.
Move of the family	25.0	Dich.	M and/or F reports that the family moved in the last 12 months.
Current or previous issues with the law	1.9	Dich.	M and/or F reports that at least one caregiver was accused, brought before the court, and/or has been incarcerated.
Mean score of familial risk factors	7.6	Cont.	Percentage of experienced familial risk factors.

*M, Mother; F, Father; Dich., Dichotomous Variable; Ord., Ordinal Variable; Cont., Continuous Variable.*

#### Emotional Problems

Primary caregivers completed the Strengths and Difficulties Questionnaire (SDQ; Goodman, 1997). Only the subscale of emotional problems (five items) was used for the present analyses. Parents were asked to rate if the five sentences were true with respect to their child on a 3-point Likert scale ranging from 1 (not true) to 3 (certainly true). A confirmatory factor analysis was carried out with data from both T2 and T3. Results indicated that longitudinal error correlations as well as two further error correlations were needed to obtain a good fit to the data, namely one representing the depressive subcomponent (i.e., items 08 and 13) and one representing the anxiety subcomponent (i.e., items 16 and 24). With these changes implemented, results supported the proposed model [χ*^2^*(25) = 26.34, *p* = 0.39, CFI = 0.99, RMSEA = 0.02, SRMR = 0.05]. McDonald’s omega reliability values were found to be 0.49 at T2 and 0.66 at T3. However, none of the five items seemed to have consistently low loadings at both T2 and T3. The item *“often complains of headaches, stomach-aches or sickness”* had loadings of 0.21 at T2 and 0.48 at T3. The two depression items *“many worries, often seems worried”* and *“often unhappy, down-hearted or tearful”* had loadings of 0.47/0.40 at T2, and 0.69/0.63 at T3 while the two anxiety items *“nervous or clingy in new situations, easily loses confidence”* and *“many fears, easily scared”* had loadings of 0.58/0.34 at T2, and 0.33/0.51 at T3. In light of the relatively small sample size on one hand, and the guideline of using exactly three indicators for each single latent variable (Little, 2013) we decided to exclude item *“often complains of headaches, stomach-aches or sickness”* and item *“many fears, easily scared”* from the present analyses, thus keeping items *“many worries, often seems worried,” “often unhappy, down-hearted or tearful,”* and *“nervous or clingy in new situations, easily loses confidence.”* The resulting model with two latent variables (T2 and T3, with three indicators each) and only longitudinal correlations fitted the data sufficiently well [χ*^2^*(5) = 9.74, *p* = 0.08, CFI = 0.91, RMSEA = 0.07, SRMR = 0.03]. McDonald’s omega reliability values were found to be 0.59 at T2 and 0.53 at T3, which confirmed the need for modeling these constructs as latent variables in further models. Descriptive statistics are reported in Table 2.

**TABLE 2:** Zero-order bivariate correlations among all study variables (*n* = 238).

		***M***	***SD***	**ICC**	**1**	**2**	**3**	**4**	**5**	**6**	**7**	**8**	**9**	**10**	**11**	**12**
1	EPa–T2	1.07	0.28	0.07	1											
2	EPb–T2	1.11	0.35	0.12	0.42***	1										
3	EPc–T2	1.55	0.63	0.14	0.25*	0.26***	1									
4	EPa–T3	1.27	0.50	0.08	0.23*	0.04	0.18*	1								
5	EPb–T3	1.14	0.40	0.05	0.22*	0.11	0.06	0.36***	1							
6	EPc–T3	1.41	0.55	0.05	0.03	0.03	0.24**	0.26**	0.18**	1						
7	SCa–T2	2.40	0.78	0.13	–0.07	–0.07	0.00	0.03	–0.07	–0.01	1					
8	SCb–T2	2.38	0.79	0.08	–0.06	–0.13	–0.02	0.05	–0.04	–0.01	0.56***	1				
9	SCc–T2	2.37	0.74	0.11	–0.05	0.05	–0.01	–0.04	0.02	0.04	0.33***	0.37***	1			
10	SEa–T2	2.71	0.53	0.07	–0.04	0.02	–0.23**	–0.07	0.05	–0.11	–0.05	–0.08	–0.11	1		
11	SEb–T2	2.94	0.56	0.08	–0.05	–0.06	–0.22**	–0.12	0.16*	–0.09	0.01	–0.05	–0.10	0.46***	1	
12	SEc–T2	2.82	0.51	0.13	–0.03	–0.07	–0.30***	–0.06	0.04	–0.10	0.02	–0.04	–0.05	0.43***	0.49***	1
13	FRF–T1	0.08	0.08	0.08	0.14^+^	0.15*	0.12	0.03	0.14	0.08	–0.11	–0.01	0.02	–0.07	–0.01	–0.01

EP, Emotional problems; SC, Positive self-concept; SE, General self-efficacy; Subscripts _*a,b,c*_, Subscripts for the different items of the various constructs; FRF, Familial risk factors; T1-T2-T3, Waves of data assessment; ICC, Intraclass correlation. ^+^*p* < 0.10;*p < 0.05; **p < 0.01; ***p < 0.001.

#### General Self-Efficacy

Primary caregivers reported on their child’s general self-efficacy by completing the questionnaire by Jerusalem and Schwarzer (1999). The questionnaire encompassed a total of ten statements describing self-efficacious behaviors and beliefs that were to be rated with respect to the child on a 4-point Likert scale ranging from 1 (not true) to 4 (true). In order to reduce the complexity of the model, the three items that met the following criteria were chosen: (1) high face validity, (2) satisfactory reliability, and (3) no problematic pattern of error correlations. The items *“In unexpected situations, my child knows what needs to be done,” “When something surprising happens, my child knows how to handle it,”* and *“When something new comes up, my child knows how to handle it”* were selected for the final model of self-efficacy. From a substantial point of view, this reduction leads to a specification of the meaning of general self-efficacy toward self-efficacy about being able to master new and surprising challenges of all kinds (i.e., with no further specification of the challenges). The resulting model with three indicators was saturated and thus fitted the data perfectly. McDonald’s omega was found to be 0.72. Descriptive statistics are reported in Table 2.

#### Positive Self-Concept

Children were administered the Self-Concept Questionnaire for Preschool (Selbstkonzept-Fragebogen im Vorschulalter; Engel et al., 2010) in form of an interview with the child that was carried out by a trained research assistant at children’s homes. The subscale *positive self-concept* encompassed a total of 15 items. For each item, children were asked if they agreed (i.e., 1 = *yes* and 0 = *no*) with the statements about themselves that were read out loud by the interviewer. If children agreed with the statement, they were asked how much they agreed with response options ranging from 1 = *a little* to 4 = *very much*. The items addressed a variety of positive self-concept facets such as knowing things, life satisfaction, liking oneself, or being able to do things. Given the heterogeneity of these items, again three items were chosen that met the criteria described above. The items *“How cool do you think you are?” “How happy are you?”* and “*How many things are you able to do?”* were selected for the final model of self-concept. These three items can be understood as closely related to life satisfaction and general ability self-concept and might reflect both actual ability and the positivity of feedback that children receive from their parents in all day situations (Eder, 1990; Harter, 1998). Again, the resulting model with three indicators was saturated and thus fitted the data perfectly. McDonald’s omega was found to be 0.70. Descriptive statistics are reported in Table 2.

### Analysis Strategy

Different strategies for the operationalization of resilience exist (Miller-Lewis et al., 2013). In the present study we followed the approach of resilience as a process in which the effect of a risk factor on a given outcome is buffered by a given protective factor. The specific modeling strategy is outlined in detail in the following.

#### Statistical Modeling Strategy

**FIGURE 1:**
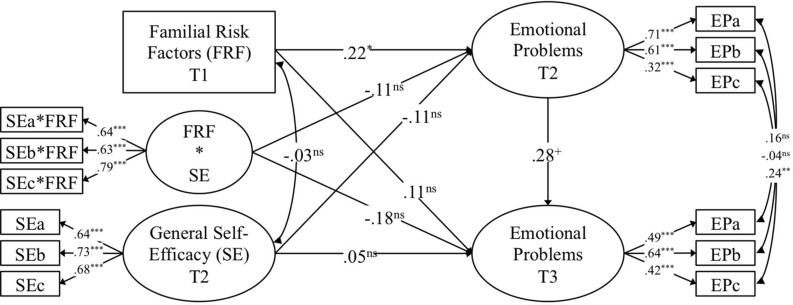
Standardized results from the model with general self-efficacy (SE) as a moderator of the association between familial risk factors (FRF) and emotional problems (EP). FRF*SE represents interaction term between familial risk factors and general self-efficacy. ns, non-significant; ^+^*p* < 0.10; *p < 0.05; **p < 0.01; ***p < 0.001.

**FIGURE 2:**
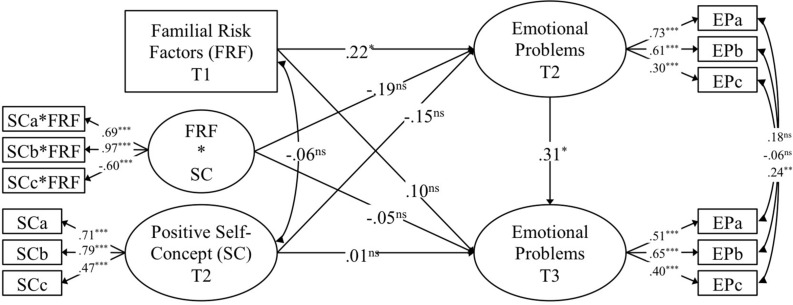
Standardized results from the model with positive self-concept (SC) as a moderator of the association between familial risk factors (FRF) and emotional problems (EP). FRF*SC represents interaction term between familial risk factors and positive self-concept. ns, non-significant; ^+^*p* < 0.10; ^∗^*p* < 0.05; ^∗∗^*p* < 0.01; ^∗∗∗^*p* < 0.001.

A series of structural equation models were used to examine the research questions at hand. Structural equation models make it possible to make use of latent variables (i.e., unobserved variables that underlie individuals’ responses to a set of items tapping into the same conceptual construct such as, for instance, general self-efficacy). The advantage of latent variables is that the amount of reliable variance can be isolated from unreliable variance (i.e., measurement error; Kline, 2016). In a first step, a latent variable for emotional problems at T2 and another latent variable for its T3 counterpart were modeled using the effect coding method^1^ and three indicators, which resulted in two saturated measurement models^2^ (Little, 2013). Herein, correlations between identical pairs of items assessed at the two measurement occasions were freely estimated since these correlations represent a shared variance among identical items over time. Further, T3 emotional problems were regressed onto T2 emotional problems. In a second step, familial risk factors (T1) were added to the model as a manifest variable (i.e., mean score of the 14 dichotomized variables assessing familial risk) and modeled as a predictor of emotional problems at both T2 (i.e., short-term effect) and T3 (i.e., long-term effect). In a third step, general self-efficacy (T2) was introduced as a latent variable (effect coded and saturated measurement model, see details above) and modeled as a further predictor of emotional problems at both T2 and T3. Additionally, general self-efficacy was allowed to correlate with familial risk factors. In a fourth and last step, we used the orthogonalization method^3^ (Little, 2013) to model a latent interaction term between familial risk factors and general self-efficacy. This latent interaction term was constrained to be uncorrelated with both familial risk factors and general self-efficacy and was then modeled as a third predictor of emotional problems at both T2 and T3. Given the complexity of the model and the comparably small sample size, we decided not to introduce the positive self-concept into the same model, but to set up an identical model in which we exchanged general self-efficacy for positive self-concept. A graphical representation of these two models can be found in Figures 1, 2. Once the two models for the two moderators were constructed, children’s sex and age were entered into the model as manifest predictors of emotional problems at both T2 and T3 and were allowed to correlate with familial risk factors, general self-efficacy or positive self-concept and their interaction (i.e., all exogenous variables in the model). Model fit evaluation was based on the conventional thresholds (e.g., Hooper et al., 2008). All models were found to fit the data well (see Table 3).

**TABLE 3:** Model fit indices of the structural equation models.

	**χ*^2^***	***df***	***p***	**CFI**	**RMSEA**	**SRMR**
**Model without control variables**						
Model for general self-efficacy	56.05	57	0.51	1.00	0.01	0.06
Model for positive self-concept	55.28	57	0.54	1.00	0.01	0.05
**Model with control variables**						
Model for general self-efficacy	71.50	73	0.53	1.00	0.01	0.06
Model for positive self-concept	71.35	73	0.53	1.00	0.01	0.05

*χ^2^, chi-square; df, degrees of freedom; p, probability of type I error; CFI, Comparative Fit Index; RMSEA, Root Mean Square Error of Approximation; SRMR, Standardized Root Mean Square Residual. Models with covariate include children’s sex and age as manifest covariates.*

#### Examination of Change in Emotional Problems

Emotional problems were assessed on two measurement occasions using the respective subscale from the Strengths and Difficulties Questionnaire (Goodman, 1997) and were modeled as a latent variable. Statements about the stability of a variable can only be made if it can be ensured that the same construct was assessed at the various measurement occasions, which is known as measurement invariance (Cheung and Rensvold, 2002). Measurement invariance was tested by comparing a configural model with unconstrained item loadings and intercepts to a metric invariance model with item loadings constrained to equality. The metric invariance model was then compared to a scalar invariance model with item intercepts constrained to equality. Table 4 shows that the metric invariance constraints did not lead to deterioration in model fit, while the scalar invariance constraints led to a strong model fit deterioration. This suggests that the way the various items represented the latent construct of emotional symptoms was stable across time, while departures of the means of the items form the means of the latent construct were not stable. This pattern of measurement invariance allows for the comparison of variances and covariances, but not of means and intercepts (Cheung and Rensvold, 2002). Given that the aim of the present study was predictive in nature and that a comparison of the means of emotional problems across time was not central, we adopted only the metric invariance constraints in further longitudinal models.

**TABLE 4:** Model fit comparison of the three models for the examination of measurement invariance.

	**χ*^2^***	***df***	***p***	**CFI**	**RMSEA**	**SRMR**	**Δχ*^2^***	**Δ*df***	***p***
Configural	9.73	5	0.083	0.91	0.07	0.03	–	–	–
Metric	10.28	7	0.174	0.94	0.05	0.04	0.54	2	0.131
Scalar	32.01	9	0.000	0.57	0.11	0.08	21.73	2	0.000

*χ^2^, chi-square; df, degrees of freedom; p, probability of type I error; CFI, Comparative Fit Index; RMSEA, Root Mean Square Error of Approximation; SRMR, Standardized Root Mean Square Residual; Δ, Difference Value.*

#### Handling of Missing Data

In order to examine the pattern of missingness over the three waves of assessment, a series of independent sample t-tests were performed. Results showed that children that participated in both T1 and T2 had slightly but not significantly lower overall scores of familial risks (β = −0.09; *p* = 0.09). As for the T2 to T3 drop out, children participating in all three assessments again had slightly but not significantly lower overall scores of familial risks (β = −0.13; *p* = 0.06). Further, they had comparable scores of emotional symptoms (β = −0.05; *p* = 0.71) and general self-efficacy (β = 0.10; *p* = 0.23) at T2 but slightly and significantly higher scores positive self-concept at T2 (β = 0.20; *p* < 0.01). These results indicate the presence of a slightly selective drop out that does, however, not seem to correlate with the target construct of emotional symptoms. Accordingly, in the structural equation models described above, the *full information maximum likelihood (FIML)* method was used to address missing under the assumption of missing at random (Schafer and Graham, 2002).

#### Handling of Nested Data

At T1, the 293 children were nested in 63 childcare groups that were in turn nested in 25 childcare centers. Accordingly, the average number of children in each childcare group was very low, which indicated that the use of a multilevel model was not indicated. However, intraclass correlation coefficients (ICC) for the nesting of children within childcare groups varied between 0.05 and 0.14 depending on the variable (see Table 2), which indicated the need to correct for resulting dependencies. To this end, we used the Huber-White sandwich estimator (Freedman, 2006). The nesting of observation within children was addressed using a longitudinal model, as explained in the statistical modeling strategy above.

## Results

### Results for Familial Risk Factors

Results pertaining to the role of familial risk factors are displayed in Figures 1, 2. As for the prediction of emotional problems at T2 (i.e., short-term perspective), familial risk factors (T1) were found to have a positive, small-to-medium, and significant effect. Further, familial risk factors were found to have a positive, small, and non-significant effect on emotional problems at T3. Thus, after controlling for children’s age and sex, children with higher scores of familial risks at T1 were found to have more emotional problems in early childhood (T2) and tended to have more emotional problems in middle childhood (T3). Considering the time lag of 1 year from T1 to T2 and of 6 years from T2 to T3 on one hand in combination with the relatively small sample size on the other hand (which manifests themselves in form of comparably small estimates and relatively large standard errors), the effects of familial risk factors, seem to be meaningful, even if effect sizes were found to be comparably small.

### Results for General Self-Efficacy

Results of the model with general self-efficacy as a moderator are displayed in Figure 1. In the short term, general self-efficacy (T2) had a negative, small, and non-significant effect on emotional problems at T2, while the interaction of familial risk factors and general self-efficacy was found to be negative, small, and non-significant. Put differently, children with higher scores in general self-efficacy tended to have somewhat lower scores on emotional problems and were found to show somewhat weaker associations between familial risk factors and emotional problems. Turning to the long-term perspective, the relative stability of children’s emotional problems was found to be of medium magnitude and non-significant: Higher scores of emotional problems in early childhood were linked to higher scores of emotional problems in middle childhood. Further, general self-efficacy had a negative, small, and non-significant effect and the interaction among familial risk factors and general self-efficacy was found to be negative, small-to-medium, and non-significant. These results suggest that, when taking children’s emotional problems in early childhood as well as their age and their sex into account, general self-efficacy in early childhood was found to have a negligible link to emotional problems in middle childhood and to slightly moderate the effects of familial risk factors on emotional problems from early to middle childhood.

### Results for Positive Self-Concept

Results of the model with positive self-concept as a moderator are displayed in Figure 2. Regarding the short-term perspective, positive self-concept (T2) had a negative, small, and non-significant effect on emotional problems at T2, while the interplay between familial risk factors and positive self-concept was found to be negative, small-to-medium, and non-significant. These results suggest that children with higher scores in positive self-concept tended to have somewhat lower scores on emotional problems and suffered less from the effects of familial risk factors on their emotional problems. From a long-term perspective, positive self-concept had an effect very close to zero on emotional problems and the interaction among familial risk factors and positive self-concept was also found to be negligible. Thus, even after controlling for children’s emotional problems in early childhood, their age, and their sex, positive self-concept in early childhood was not found to have a substantial link to emotional problems in middle childhood or to protect from the effects of familial risk factors on emotional problems from early to middle childhood.

### Results Pertaining to Covariates and Additional Results

Familial risk factors were found to be virtually uncorrelated to both general self-efficacy (*r* = −0.03; *p* = 0.78) and positive self-concept (*r* = −0.06; *p* = 0.49). Children’s sex and age were found to be uncorrelated (*r* = 0.04/0.04; *p* = 0.55/0.57)^4^. Moreover, children’s sex was not found to have a meaningful link to familial risk factors (β = −0.01/0.01; *p* = 0.92/0.92), their general self-efficacy (β = −0.06; *p* = 0.45), or their positive self-concept (β = 0.01; *p* = 0.92), and was not found to have a relevant association with their emotional problems at T2 (β = 0.01/0.02; *p* = 0.92/0.84) or T3 (even after controlling for emotional problems at T2; β = 0.05/0.05; *p* = 0.64/0.62). Similarly, children’s age was found to have a weak link to familial risk factors (β = −0.04/−0.04; *p* = 0.56/0.58), their general self-efficacy (β = 0.07; *p* = 0.43), or their positive self-concept (β = 0.08; *p* = 0.30), and was found to have an negligible effect on their emotional problems at T2 (β = −0.07/−0.04; *p* = 0.53/0.69) or T3 (even after controlling for emotional problems at T2; β = 0.04/0.05; *p* = 0.71/0.59).

## Discussion

The present study examined the promotive and protective role of general self-efficacy and positive self-concept in the context of potential undesirable effects of cumulated early familial risk factors on children’s development of emotional problems from early to middle childhood. Results will be discussed starting from the stability of emotional problems from early to middle childhood, followed by the role of familial risk factors, and general self-efficacy as well as positive self-concept as promotive and protective factors.

### Stability of Emotional Problems

On the first measurement occasions, children were between 3 and 5 years old, while in the second one they were 9 to 11 years old, thus resulting in a time lag of roughly 6 years. The autoregressive effect of emotional problems was found to be of medium size (Cohen, 1992) with an effect of roughly β = 0.30 and to oscillate around the threshold of significance depending on the constellation of the other variables in the model. This result suggests that the amount of variance of the T3 score of emotional problems that can be explained by the T2 score of emotional problems is around 10%, thus leaving a large unexplained portion. Two conclusions can be drawn from these findings. First, the statistical power of the analyses at hand seems to be somewhat low given that medium effects struggle to reach statistical significance. Consequently, an even stronger focus on effects sizes instead of significances is in order. Indeed, as Cohen (1990) clearly stated in his seminal work, research should focus on reporting effect sizes and on comparing them to effect sizes reported in other studies on the same or similar topics. Second, taking the time gap of 6 years into account, the relative stability (i.e., the ordering of children from the lowest to the highest score) of emotional problems from early to middle childhood might be seen as moderate. As a comparison Bandura et al. (1999) found a relative stability over 1 year in early adolescence of β = 0.40. Given the high fluctuation in emotional problems during childhood, the question regarding the factors that underlie this variability arises. In this regard, the focus of the present study was set on the interplay among familial risk factors and two aspects of children’s self-referential mental representations, namely general self-efficacy and positive self-concept. The respective results are discussed in the following.

### The Role of Familial Risk Factors

In keeping with studies showing that dose-response effects of risk factors on child development (Liming and Grube, 2018), familial risk factors were operationalized as a cumulative score of 14 factors ranging from comparably distal risk factors such as poverty or single-parent family, to comparably proximal ones such as chronic disharmony or violence in the family. Descriptive results showed that the prevalence of the 14 familial risk factors varied from quite low rates (e.g., serious illness or death of a friend with a prevalence rate of 1%) to quite high rates (e.g., move of the family with 25%). These results confirm that the sample that took part in the present study was a community sample as opposed to a high-risk sample.

As for the effect of familial risk factors on emotional problems, a positive effect was found both in the short-term, which confirms hypothesis 1, and in the long-term perspective. This pattern of results aligns with previous studies and the size of the effects of familial risk factors on child outcomes is comparable to those found in previous studies: Nguyen and Scott (2013) found a cross-sectional standardized effect of death of a family member on children’ depression in Grades 2–6 of β = 0.10 (rounding up). Parental distress was also found to have a small-to-medium effect of β = 0.21 on children’s internalizing problems in a cross-sectional studies among children and adolescents aged 5–18 (Annunziato et al., 2007). In a longitudinal study across preschool, the effect size of children’s early familial risks (as a composite score of biological, economic, human capital, and demographic variables) showed a weak and non-significant association of β = −0.08 (see results of Model 4) association with numeracy skills at the end of preschool (Kluczniok, 2017). Further, Ackerman et al. (2002) were able to show that, for instance, chronic instability (β = 0.20), family income (β = −0.26) and recent maladjustment (β = 0.19) were linked to third-graders internalizing problems. In light of the magnitude of these effect sizes, the short- and long-term associations between familial risk factors and emotional problems that were found in the present study seems to be comparably high, which underlines their relevance.

Three additional considerations about the study design are relevant for a contextualized interpretation of the present results: (1) The time lag between the assessment of familial risk factors and emotional problems needs to be taken into account. In the short-term model, the time lag was about 1 year, which is not necessarily a short period of time. In line with this thought, the roughly 7 years that separated the assessment of familial risk factors from the assessment of emotional problems in middle childhood can be regarded as a comparably long time lag. Given previous studies showing that the effect of risk factors tends to become weaker with time (Korol et al., 2002), the effect size might be interpreted as relevant; (2) It must be considered that the sample size of the present analyses was comparably low from a purely statistical perspective, which directly influenced the power to detect small effects; (3) The complexity of the operationalization of family risks prohibited its inclusion in the model as a latent variable, particularly with the aim to include it in the computation of a latent interaction term. Therefore, familial risk factors included a certain extent of measurement error, which might have resulted in an underestimation of the effects as well as of their statistical significance. These aspects being considered, we cautiously conclude that familial risk factors were linked to emotional problems both in the short- and long-term even though future studies among larger samples and more measurement occasions might be needed to confirm this result.

### The Role of General Self-Efficacy

Before results about the promotive and protective role of general self-efficacy are discussed, it is worth noting that results indicated that the effect size of the association between general self-efficacy and familial risk factors was very close to zero and non-significant. This result gives some tentative credit to the notion of general self-efficacy as a moderator (i.e., a variable that is *un*correlated with the risk factor and that affects the relationship between a risk factor and an outcome variable) as opposed to a mediator (i.e., a variable that is caused by the risk factor and in turn affects the outcome variable) in early childhood. Accordingly, the role of general self-efficacy as a moderator will be discussed in the following.

Children’s general self-efficacy was found to have a negative main effect of small magnitude on emotional problems in the short-term perspective. This effect was not found to be statistically significant, which on one hand suggests that the effect might be non-existent but on the other hand also indicates that the statistical power of the analyses at hand might not be the strongest when taking family risk factors, age, and gender into account. Thus, the short-term results are not in line with hypothesis H2a. Nevertheless, it might be argued that the short-term effect size at hand cannot be discarded as irrelevant since it is at least small. The lack of studies with a similar focus and design as the present study makes it hard to estimate whether the effect size of self-efficacy must be considered as comparable or different to other studies. Nonetheless, among a sample of early adolescents, Bandura et al. (1999) found a longitudinal effect of academic and social self-efficacy of a magnitude between β = −0.14 and −0.30. Accordingly, our results seem to be at the lower end of strengths of associations between general self-efficacy and emotional problems. This result could be explained by the focus on general self-efficacy as a broad construct as opposed to more task-specific forms of self-efficacy.

Turning to the moderation effect of general self-efficacy on the association between familial risk factors and emotional problems, a small but non-significant buffering effect was found, which leads to the rejection of hypothesis H3a. Given that moderations analyses are chronically underpowered (Aguinis et al., 2005), this small effect in combination with the small main effect suggest that children with higher scores in general self-efficacy seem to have slightly lower scores on emotional problems in early childhood and to suffer less from the effects of familial risk factors on their emotional problems. An examination of the magnitude of the main and moderating effect of general self-efficacy suggests that the effect of familial risk factors can be almost entirely compensated by general self-efficacy. This pattern of results is in line with theoretical assumptions stemming from the social-cognitive theory (Bandura, 1977).

As for the longitudinal role of general self-efficacy, the long-term main effect was found to be very close to zero while the moderation effect was actually found to be negative (i.e., protective) and of small-to-medium magnitude. Again, both effects were not found to be statistically significant. While regarding the main effect it could be argued that the large time lag between early and middle childhood reduced the effects of general self-efficacy, the moderating effect seems to be still noticeable. However, the interpretation of the moderation effect is somewhat inconsistent in the absence of a main effect, since the pattern at hand suggests that for children with low scores on familial risk factors, higher scores of general self-efficacy are linked to more emotional symptoms (although the overall level would be still lower than for most children with high levels of familial risks). Conversely, for children with high scores on familial risk factors, higher scores of general self-efficacy are linked to less emotional symptoms (although the overall level would be still higher than for most children with low levels of familial risks). Based on this incomplete pattern, we conclude that the longitudinal protective and promotive role of self-efficacy are very weak and would need replication in order to allow for a stable interpretation.

In conclusion, general self-efficacy seems to play a promotive as well as a protective role in the short-term, although the magnitude of the effects is rather small. In the long-term, the promotive role of self-efficacy seems to be weak and difficult to grasp as the absence of a promotive effect makes the logic of a protective effect somewhat questionable in this very specific context.

### The Role of Positive Self-Concept

In keeping with results reported above for general self-efficacy, positive self-concept was also not found to have a relevant or significant link to familial risk factors in early adolescence. Therefore, positive self-concept seems to be more of a moderator (Nguyen and Scott, 2013) than a mediator in the conceptual constellation present study, while studies with a different framework worked with self-concept as a mediator (Spilt et al., 2014).

From a short-term perspective, positive self-concept was found to have a weak although non-significant promotive and a small-to-medium but non-significant protective role in the present study. While the non-significance of these results imply the rejection of hypotheses H2b and H3b, the magnitude of the effects might justify interpreting these results as indications of a meaningful role of positive self-concept. Indeed, it could be argued that the sum of the promotive and the protective role of positive self-concept are such that the undesirable effects of familial risks can be more than compensated in the short term. Further, as a comparison, Nguyen and Scott (2013) found a main effect of physical self-concept on children’s depression of β = −0.03 (non-significant) and a protective effect on the link between the death of a family member and children’s depression of β = −0.12 (*p* < 0.05). Our results thus seem to be in line with previous research as the effects seems to be comparable.

Regarding the long-term perspective, no indications of a promotive or protective role of positive self-concept were found in the present study. This suggests that the already comparably weak effect of positive self-concept in the short term diminishes over time.

### Implications for Practice

Masten and Barnes (2018) summarized three basic strategies that foster positive development across the lifespan: (1) Early identification and reduction of risk factors, (2) foster promotive and protective factors, and (3) activate the entire system to develop its sources of resilience. Regarding the reduction of familial risks, the main sources of the threat of a system need to be identified and strategies need to be developed to tackle them in a way that reaches the entire population. Examples here include measures to tackle poverty, to reduce the incidence of maternal depression, to augment work-family balance, to screen for early predictors of maladaptive development in physical and mental health and to create institutions that offer affordable help for families and individual in need without stigmatizing them. Turning to the point about boosting promotive and protective processes, the present results as well as results from other studies suggest that measures aimed at improving self-efficacy and/or self-concept (and other forms of self-representations) might be beneficial in the short term both in a promotive and a protective manner. Since our results suggest that the desirable effects of self-representations are short-termed, it seems to be important to work with strategies that offer a sustainable support over prolonged periods of time, ideally such that they reinforce themselves.

Given the complex nature of resilience processes, it seems to be most promising to tackle the issue of children’s emotional problems by addressing as many levels of a system as possible. Also, it is important to take advantage of the different windows of occasions that have been documented in research on resilience (Masten and Barnes, 2018). Caregivers, educators, teachers, peers, and policy makers must work together to ensure that strategies can be developed that work as intended and that reach the individuals that need the most: families with cumulated risks.

### Strengths and Limitations

To the best of our knowledge, this was the first study to examine the promotive and protective role of general self-efficacy and positive self-concept in the context of maladaptive effects of early familial risks on children’s development of emotional symptoms from early to middle childhood. The main strengths of this contribution lie in the longitudinal perspective with a starting point in early childhood as well as the use of sophisticated statistical methods for the simultaneous analysis of promotive and protective effects, which allowed to tackle some critical aspects discussed in the limitations below. Other strengths are the combination of 14 familial risk factors with two promotive/protective factors, one as reported by caregivers and one as reported by the children themselves. This combination helped reduce the common method bias.

On the limitations side of things, it is important to highlight that most measures used in this study were subjective in the sense that they were gathered using self- or other reports, which might have caused a certain level of common method bias. At the same time, the inclusion of child reports can be seen as a strength considering that it adds the perspective of the child and reduces common methods bias. Additionally, we were not able to compare the means of the two measurement of emotional problems due to the absence of scalar invariance. Moreover, the reliability of the emotional problems subscales was found to be somewhat low at both measurement occasions, which was addressed using latent variables. Finally, the large time gap between T2 and T3 in combination with the relatively small sample size led to small effects with large standard errors, which directly translated into a higher likelihood to obtain non-significant results. Nevertheless, there are other potential causes for non-significant and/or small effects. One main reason is that we have used a community-based sample, which leads to low risk scores and to a low variability thereof. From a statistical point of view, the fact that distributions are very skewed also poses a limitation to the ability to detect effects. Similar issues have been reported in studies with high risk samples (Markson et al., 2016). An oversampling of families with high risk might be indicated to generate more variance both in terms of risk as well as in terms of outcomes and moderators, which might lead to a better visibility of statistical effects (i.e., larger effect size). Moreover, a weakness of the assessment of familial risk factors as assessed in this study is that it might not represent a true cumulative risk factor, as it lacks information on the severity of the various risk factors and on their actual accumulation in terms of additive associations with the outcome of interest. Also, multiplicative associations cannot be examined with the approach chosen here. Another reason might be the fact that the variables at hand are not necessarily directly linked. For instance, the link between parental drug consumption and emotional symptoms might be indirect and involve a number of behavioral variables such as reduced parent-child interaction quality and emotional availability that might be caused by increased drug consumption. A further source of bias might be a limited validity and reliability of the measures that are typically used. Self-report measures might suffer from psychometric weaknesses that might be caused by social desirability, recall-bias, misinterpretations, or even fatigue effects in long questionnaires. Further, the timing of assessment might be crucial in terms of the potential to detect the expected effects, in particular for risk factors that are acute rather than chronic: For example, the effects of witnessing violence or conflicts in the family might be less constant than those of low maternal education and the visibility of effects of the former might thus depend more on the timing of assessment than for the latter. In sum, future studies might take advantage of more assessments with shorter time gaps and of larger sample sized in order to deepen our knowledge about promotive and protective mechanisms in the context of resilience across childhood.

## Data Availability Statement

The original contributions presented in the study are included in the article/Supplementary Material, further inquiries can be directed to the corresponding author.

## Ethics Statement

Ethical review and approval was not required for the study on human participants in accordance with the local legislation and institutional requirements. Written informed consent to participate in this study was provided by the participants’ legal guardian/next of kin.

## Author Contributions

FS was responsible for the entire project data management, analyzed and interpreted the data, and wrote the first draft of the manuscript for the present study with valuable feedback from CW and OG-H. CW acquired the funding, designed and managed the study, collected and/or developed the instruments, and recruited the sample. OG-H helped to recruit the sample, organized the data collection and collected the data together with a number of undergraduate students. FS, CW, and OG-H have critically reviewed and revised the manuscript. All authors have read the final version of the manuscript and agreed to publish it in its present form.

## Conflict of Interest

The authors declare that the research was conducted in the absence of any commercial or financial relationships that could be construed as a potential conflict of interest.

## Footnotes

1 The advantage of effect coding over other methods of latent variable identification is that it yields a more balanced representations of all indicators that are used to model a latent variable, instead of over-representing an arbitrarily chosen reference indicator (Little, 2013).

2 A latent variable that is modeled with exactly three indicators yields a so called saturated (or just-identified) measurement model for the latent variable at hand. This strategy comes with the advantage that the measurement models does not generate degrees of freedom that artificially increase the fit of the entire structural model (Little, 2013).

3 This technique makes it possible to model a latent interaction term that is completely uncorrelated (i.e., orthogonal) to the latent variables that were used to compute it. Thus, the latent interaction term is also free of measurement error and does not change the interpretation of the other variables’ main effects, which facilitates the interpretation of the results (Little, 2013).

4 The first value stems from the model with general self-efficacy as a moderator, while the second value stems from the model with positive self-concept as a moderator.

## Supplementary Material

Supplementary Data Sheet 1 Original data.
